# Monitoring of Strain and Temperature in an Open Pit Using Brillouin Distributed Optical Fiber Sensors

**DOI:** 10.3390/s20071924

**Published:** 2020-03-30

**Authors:** Chiara Lanciano, Riccardo Salvini

**Affiliations:** Earth and Physical Sciences and Centre for Geotechnologies CGT, Department of Environment, University of Siena, Via Vetri Vecchi 34, 52027 San Giovanni Valdarno (AR), Italy

**Keywords:** marble quarry, distributed optical fiber sensors, brillouin shift frequency, strain, temperature, unmanned aerial vehicle, robotic total station, geotechnical monitoring system

## Abstract

Marble quarries are quite dangerous environments in which rock falls may occur. As many workers operate in these sites, it is necessary to deal with the matter of safety at work, checking and monitoring the stability conditions of the rock mass. In this paper, some results of an innovative analysis method are shown. It is based on the combination of Distributed Optical Fiber Sensors (DOFS), digital photogrammetry through Unmanned Aerial Vehicle (UAV), topographic, and geotechnical monitoring systems. Although DOFS are currently widely used for studying infrastructures, buildings and landslides, their use in rock marble quarries represents an element of peculiarity. The complex morphologies and the intense temperature range that characterize this environment make this application original. The selected test site is the Lorano open pit which is located in the Apuan Alps (Italy); here, a monitoring system consisting of extensometers, crackmeters, clinometers and a Robotic Total Station has been operating since 2012. From DOFS measurements, strain and temperature values were obtained and validated with displacement data from topographic and geotechnical instruments. These results may provide useful fundamental indications about the rock mass stability for the safety at work and the long-term planning of mining activities.

## 1. Introduction

Due to its geological characteristics, in Italy, extraction sites are widely diffused in all regions [1]. In particular, the Tuscany Region is characterized by the presence of marble quarries that represent the strong economic activity for the area. The only province of Massa-Carrara in the year 2016 extracted 844,000 tons of marble blocks [2].

Despite their economic importance, marble quarries are quite dangerous environments in which rock falls and accidents may occur. As many workers operate in these sites, it is necessary to deal with the issue of safety at work. The use of innovative technologies and the analysis of the stability of quarry slopes can contribute to improving the conditions of safety in the workplace.

To obtain information about the status of the rock mass and the slope stability, it is necessary to measure data that characterize the observed site as the properties of rock mass and joint systems. There are several methods to acquire this type of data; the most widespread include classical structural and engineering-geological surveys, geotechnical sensors (ex. extensometers, crackmeters), ground-based radar interferometry, GNSS (Global Navigation Satellite System) and Robotic Total Station. In this paper, an innovative monitoring system developed as part of the R&D project POR FESR 2014-2020, named "Real-time monitoring of quarry walls using fiber optic sensors" [3,4], is shown. The project was led by the “Cooperativa Cavatori Lorano” (Carrara, Italy) and came about from the collaboration with the “Centre of Geotechnologies” (CGT) of Siena University (Italy) and “Geo Explorer S.r.l”, a start-up society of Siena University, which is part of the Regional Technological Marble District. The project partnership also consisted of the “Cooperativa Apuana Vagli di Sopra” (Lucca, Italy) and the “Cooperativa Levigliani” (Lucca), managers of another open pit and of an underground marble quarry, respectively, where the use of DOFS was also experimented. The project planned activities concerned the implementation of a monitoring system of the potentially unstable marble quarry fronts using DOFS. The aim of this study was to develop a more efficient and well spatially distributed system, compared to traditional surveillance techniques, capable of advising movements of the rock mass as indicators of changed stability condition and sources of potential risk. 

The recent literature reports on application of fiber optic networks in mines to monitor the structural integrity, environmental safety and production parameters. Fiber optic is capable of seismic event and mine pressure detection, methane gas monitoring, temperature monitoring and water pressure monitoring in a way to provide information for accident prediction and early warning. Liu et al. described an all fiber optic comprehensive underground coal mine safety monitoring system in China [5]. Already in 2007, Naruse et al. presented a work on the installation of an underground mine monitoring system based on a fiber optic system in the El Teniente mine (Chile) aiming to monitor the deformation due to mining activities. The monitoring system consisted of optical fiber sensors attached to the tunnel ceiling and sidewalls using rock bolts [6]. Bin and Hua presented a paper on Brillouin Optical Time Domain Reflectometry (BOTDR) using sensors installed in boreholes to monitor rock deformation within an excavated roadway in Zhangji coal mine, China [7]. Zhao et al. proposed a displacement monitoring methodology of rock layers overlying a coal seam in Zhu Xian-zhuang mine (China) based on fiber Bragg grating displacement sensors [8]. Cheng et al. measured the deformation of overlying rock layers of a coal seam by employing a BOTDR-based monitoring method [9]. Wang and Luan built a fiber mesh structure on mine roof and conducted BOTDR strain measurement [10]. Zhigang et al. conducted an experimental study using fiber optic sensing on the monitoring of deformation in the shallow layers of waste rock from the mining process in the Chinese Nanfen open pit iron mine [11]. Arzu et al. built up a laboratory experiment set-up containing an optical fiber system to simulate landslide phenomenon and to record movements [12]. 

In Italy, Matano et al. reported the implementation of an integrated system aimed at controlling the rock slope stability in the Coroglio tuff cliff, located in the highly urbanized coastal area of Naples at the border of the active volcanic caldera of Campi Flegrei [13]. Schenato et al. used a distributed optical fiber sensing system to measure landslide-induced strains on an optical fiber buried in a large-scale physical model of a slope [14].

**Figure 1:**
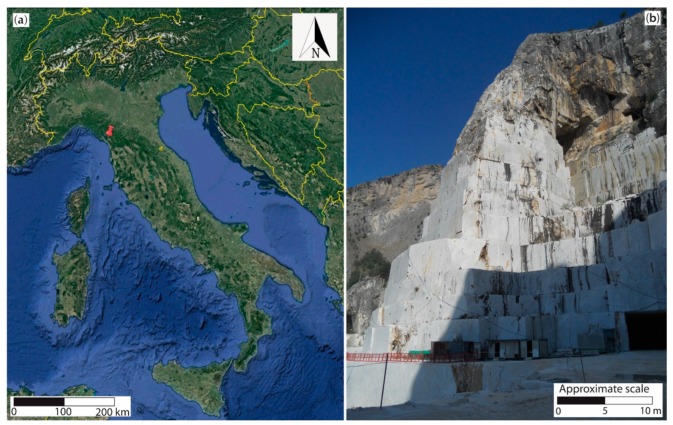
(**a**) Location of the Lorano marble quarry in Italy. (**b**) Panoramic image of the investigated Pradetto cut site.

In this work, among the three available sites of the R&D project, the Lorano “I” N° 22 quarry (Pradetto site—Figure 1) was selected to describe DOFS monitoring system results. In this quarry, an integrated topographic-geotechnical monitoring system, constituted by a Robotic Total Station (RTS), measuring every day several prisms, extensometers, crackmeters and clinometers, has been active since 2012 [15]. Therefore, thanks to this configuration, it was theoretically possible to compare new DOFS results with data acquired by the other techniques already widely discussed in [15]. The data presented in this paper refer to the year 2018.

The test site, as already said, is located in the Apuan Alps, a mountain range in northern Tuscany (Italy, Figure 1a) delimited by the following natural boundaries: the Serchio River to the NE and SE, the Aulella River to the N, the Magra River to the NW and the Versilia coastal plain between the Magra and Serchio rivers to the SW. The name “Alps” is due to a very typical alpine appearance consisting of high peaks, narrow ridges and deep-cut valleys. These mountains represent the most important tectonic window of the Apennine chain, a fold and thrust belt produced by the convergence of the African plate towards the European one [16,17,18,19,20,21,22]. First described by [23], the Apuan Alps complex, composed by the Massa and Apuan tectono-metamorphic units, is interpreted as result of two main tectonic phases known as “D1” and “D2” [15,24,25,26,27,28]. The first is a ductile compressional event (late Oligocene-very early Miocene), which originated from a progressive deformation with intense foliation [29]. The second, a ductile extensional occurrence dating back to the early Miocene, produced both folds and high-strain shear zones [29].

The Lorano open pit, which falls within the Apuan Unit, where the Upper Triassic–Oligocene metasedimentary sequence overlaps the Palaeozoic basement [15,24,25], is located in the normal limb of the “Pianza anticline”. The latter and the “Vallini syncline” form an antiform–synform pair marked by a core of Jurassic marbles and cherty meta-limestones; these are minor folds (hectometer-scale) between the “Carrara syncline” and the “Vinca anticline”, structures that can be referred to as the D1 phase [15,30]. 

The study area belongs to the Torano marble extractive basin, where there are several active open pits with quarry walls that can reach hundreds of meters in height: the landscape is therefore characterized by natural and anthropic slopes giving a very complex morphology. 

The dominant variety of marble in the Lorano quarry is the “White Marble” (about 100–200 μm grain size), with colors varying from white to ivory–white and pearl–white to light grey [31]. Moreover, the “Ordinary Marble” (about 200 μm grain size) characterized by colors from pearl–white to light grey [32] and two subordinate categories, the “Veined Grey Marble” and the “Breached Marble” [15,33], outcrop.

A typical Mediterranean climate, with hot dry summers and cold wet winters, affects the quarry area; the copious rainfall (over 3000 mm yr−1) shows a primary maximum value in the autumn season and two secondary peaks in winter and spring [15,34]. 

A very important element of the Lorano open pit is the Pradetto cut site (Figure 1b), a marble buttress (about 120 m high, 30 m wide and 40 m deep) derived from previous mining activities and object of monitoring activities as described in [35]. Accessible from three sides, at its base, the excavations keep on with a downward feed. While the “Ordinary marble’’ outcrops in the buttress, the ‘‘Veined Grey Marble’’ characterizes the mountain above. Regarding the structural and engineering-geological analysis, previous studies [15,35,36] show four high angle sets of discontinuities and, despite a good quality of the rock mass (from the Basic Rock Mass Rating RMR_b_ [37]), potentially unstable joint systems along the three different slopes of the buttress were highlighted from kinematic stability analyses [15]. 

## 2. Materials and Methods

### 2.1. UAV Photogrammetry 

**Figure 2:**
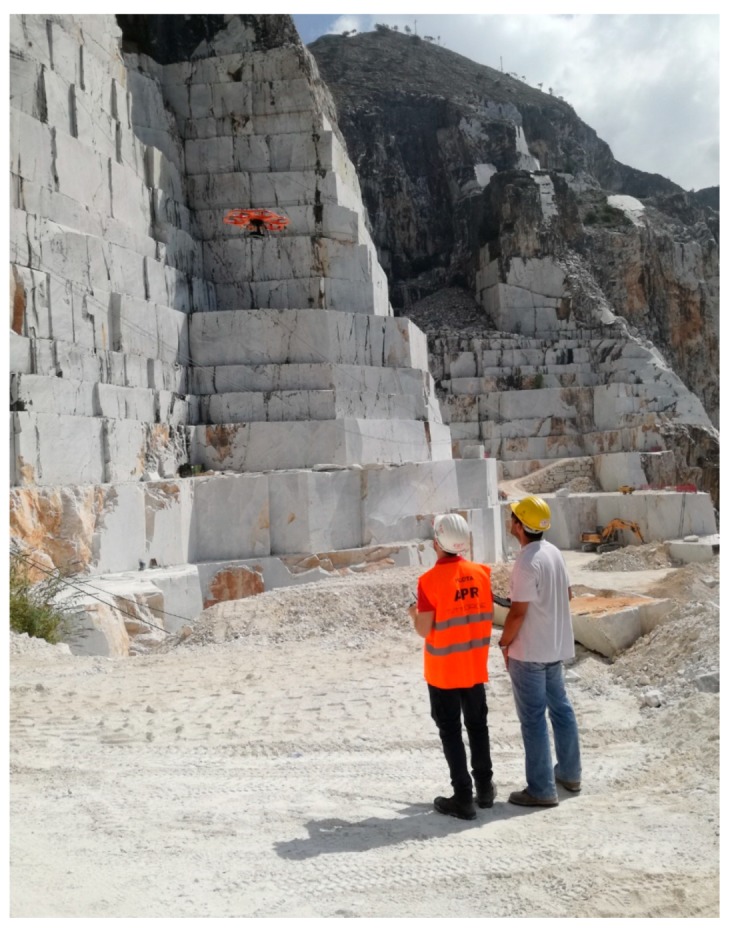
UAV survey at the Lorano marble quarry.

Under the guidance of previous results on slopes stability, DOFS were installed on the buttress by specialized climbing workers. With the scope of determining and georeferencing the DOFS exact position and facilitating comparisons with the topographic-geotechnical monitoring system, an aerial photogrammetric survey was carried out through an Unmanned Aerial Vehicle (UAV). The photogrammetric survey was carried out using the Aibotix^TM^ X6 V1 multirotor drone (Figure 2) which, with an autonomy of about 15 minutes, can operate in the visible range (400-700 nm) of the electromagnetic spectrum using a Nikon Coolpix A type camera. The equipment also consists of i) the Inertial Navigation System (INS) with GNSS, accelerometers and gyroscopes, ii) a video camera for remote inspection and iii) the flight management software. The flight was designed defining a Ground Sampling Distance (GSD) of about 1.4 cm/pix and an average flight distance from the slopes of about 50 m. As Ground Control Points (GCPs), which are necessary to improve the accuracy of the exterior orientation of the photographs, and Check Points (CPs), whose function is positional accuracy assessment, natural and artificial targets of known coordinates taken from previous works [35] (i.e., aerial photogrammetric surveys and terrestrial laser scanning) were used. For this reason, it was not necessary to perform a new topographic survey.

Photogrammetric data processing was performed using the code Agisoft^TM^ Metashape Professional which is based on the “Structure from Motion” (SfM) technique. This is a "range imaging" methodology, belonging to the “computer vision” and the visual perception [38,39,40,41,42], aimed at the reconstruction of three-dimensional structures starting from sequences of two-dimensional images. The software uses robust algorithms which allow to adjust the orientation of the frames and generate three-dimensional georeferenced and scaled point clouds, Digital Elevation Models (DEMs), three-dimensional mesh-like models and orthophotos of the area of interest.

For this case study, the image processing involved 548 digital photographs and the positioning of 49 GCPs and 8 CPs. The final Root Mean Square Error (RMSE) of the exterior orientation phase is around 8 cm for GCPs and 10 cm for CPs.

### 2.2. Topographic Monitoring System

**Figure 3:**
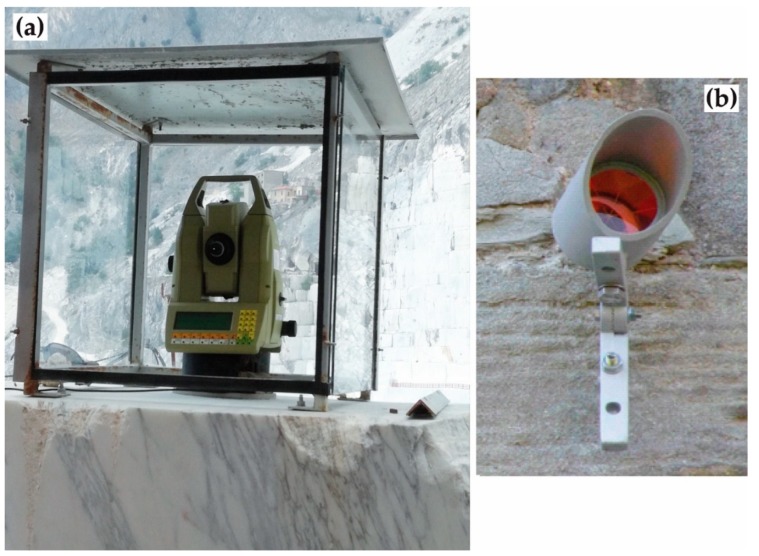
(**a**) RTS installed at the Lorano marble quarry. (**b**) Detail of a prism placed on the excavation site walls.

The topographic monitoring system installed at the open pit consists of a Leica^TM^ TCA2003 RTS (Figure 3a), which is a tool for topographic survey that combines a laser distancemeter, an electronic theodolite and a computer on a single device. More details about the instrument can be found in [15].

**Figure 4:**
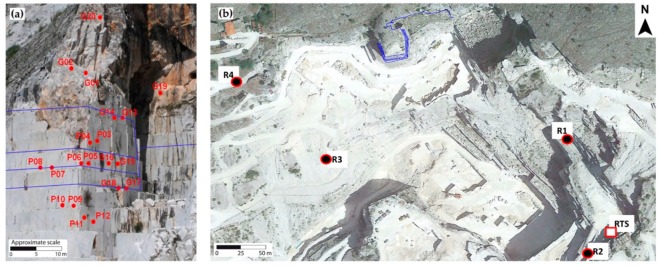
(**a**) Perspective photograph of the marble buttress with RTS measurement prisms (red points) and the DOFS trace (blue lines). (**b**) UAV-orthophoto showing the position of RTS, reference prisms (black and red points) and fiber optics (blue lines).

At the Lorano site, the RTS was placed on top of a stable marble block [15], at an approximately 300 m distance from the buttress and it was protected by a metallic box with anti-aberration glasses (Figure 3a). The RTS, starting from December 1st 2012, automatically detects, every 6 h (at 0.00, 06.00, 12.00 and 18.00 h), the 3D distance measurement of 24 prisms (an example in Figure 3b) positioned both on the pillar (20 measurement points) and outside it (4 reference points), so as to be able to discriminate between the relative movements due to local fracturing from the absolute ones, i.e., those of the entire pillar. The reference points (Figure 4), moreover, allow to have additional control in external stable areas, useful, above all, in the calibration phase of the monitoring system.

The transfer of the acquired data takes place in real-time through a telephone line that links the RTS and the CGT, where a PC controls the whole system functioning and data storage and processing. Commercial software packages (i.e., GeoMoS Monitor from Leica™, Analysis from Geodesia™ and System Anywhere from Geodesia™) control the RTS, process the data and produce instantaneous time-displacement graphs.

### 2.3. Geotechnical Monitoring System

**Figure 5:**
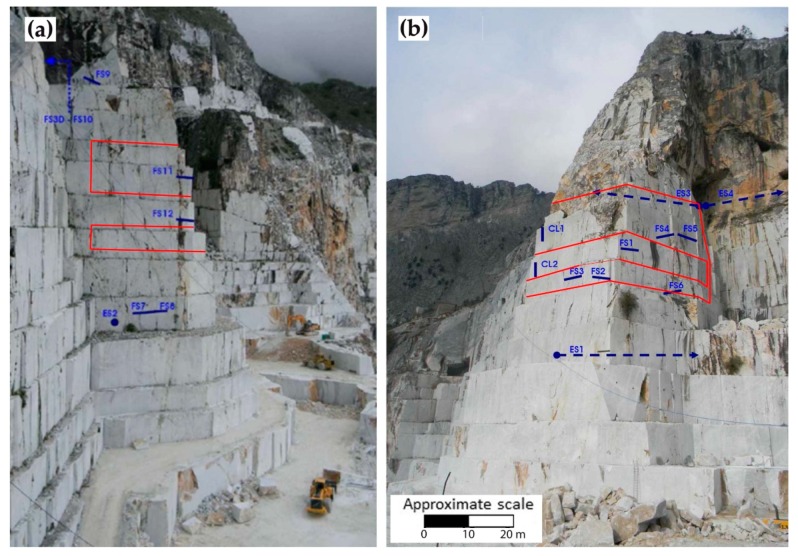
Geotechnical sensors (in blue) and fiber optics cable (in red) on the western (**a**) and southern-eastern (**b**) sides of the buttress.

The geotechnical monitoring system of the marble buttress consists of 12 monoaxial mechanical crackmeters (FS_n_ in Figure 5), 1 three-directional crackmeter (FS3D), 2 biaxial clinometers (CL_n_) and 4 multipoint borehole extensometers (ES_n_), the deepest of which was placed at a depth of 30 m [15,43,44]. This system has been operative since July 27, 2012, providing high-temporal-frequency deformation trends to be compared with seasonal variations in the climatological data (rainfall and temperature) and data from the RTS [15].

### 2.4. DOFS Monitoring System

Sensors based on optical fibers [45,46,47,48,49,50,51,52,53,54,55,56,57,58] are currently widely used for monitoring infrastructures, bridges, dams, buildings and landslides with many inherent advantages [45] including i) resistance to electromagnetic interference, ii) light weight, iii) small size, iv) high sensitivity, v) high-temperature performance, vi) immunity to corrosion and vii) large bandwidth [46].

DOFS are cables of optical fiber which offer the possibility of monitoring variations of one-dimensional structural physical fields along the entire optical fiber in a truly distributed way [51,52]. 

Among the different types of DOFS, Brillouin scattering-based sensors allow to obtain distributed strain and temperature measurements by detecting a frequency shift [50]. Specifically, Brillouin scattering is a process of diffusion of acoustic light thermally generated in the optical medium; due to the Doppler effect, the diffused light presents, with respect to the incident light wave, a frequency shift $\nu_{B}$ named “Brillouin Frequency Shift” (BFS):

(1) $$\nu_{B}=\frac{2nV_{a}}{\lambda_{0}}$$

where *n* is the effective refractive index, *λ_0_* the wavelength of the incident light in the void and $V_{a}$ the velocity of the acoustic wave [53]. The BFS is a parameter related to the optical and elastic properties of the fiber [54] and it depends on both material temperature and density; moreover, the elastic properties of silica (glass fibers) are such that an induced strain causes a change in volume and therefore, a variation in the material density. Then, any local variation of temperature and/or fiber strain, acting on the acoustic speed, produces a variation in the local value of the BFS [55]. Experimental measurements show an excellent linearity in Brillouin shift dependence on strain and temperature [54]. The relationship between the variations of strain (∆ε), temperature (∆T) and BFS (∆ $\nu_{B}\;$) is described as [53]

(2) $$\Delta \nu_{B}\left(\varepsilon ∕T\right)=C_{T}\Delta T+C_{\varepsilon }\Delta \varepsilon$$

where $C_{T}$ = (1.26 MHz/°C) and $C_{\varepsilon }$ = (0.06 MHz/με) are, respectively, the temperature and strain coefficients at 1550 nm for a single mode silica fiber [53] (the coefficients values change slightly for different types of single mode fibers). In particular, the fiber used at the Lorano site was calibrated in laboratory in order to obtain the calibration coefficients; the following temperature and strain coefficients were obtained: *C_T_* = 1 MHz/°C and *C_ɛ_* = 0.05 MHz/με.

**Figure 6:**
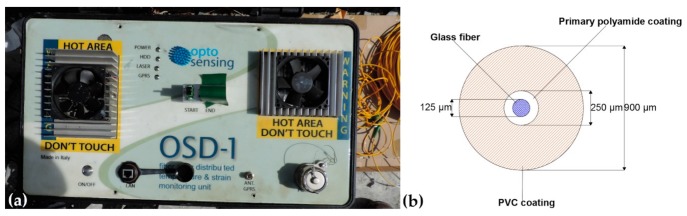
(**a**) Photograph of the OSD-1 measurement unit. (**b**) Cross section of the installed optical fiber sensor with the three different layers (glass fiber, primary polyamide coating and polyvinyl chloride coating) and their dimensions.

Sensors based on Brillouin scattering can be classified into two main types: sensors based on spontaneous Brillouin scattering, in which only the incident light is launched into the optical fiber, and sensors based on stimulated Brillouin scattering (SBS), characterized by an additional stimulation on the generation of phonons [56]. Among the techniques based on SBS, the Brillouin Optical Time Domain Analyse (BOTDA) methodology [46,52,54,56], due to its powerful signal and spatial resolution, was considered suitable and, therefore, adopted for monitoring the buttress at the Lorano marble quarry. Data were obtained using the OSD-1 system [58] (Figure 6a) provided by the company Optosensing S.r.l. The OSD-1 system, which was periodically checked both on site and remotely via PC, is a control unit able to send the logs directly to a centralized enterprise-type database. Logs provide distance from the source and microstrain values that are archived into the database in a georeferenced mode.

After acquiring the BFS, the strain profile between two consecutive measurements is given by

(3) $$S\left(z\right)=\left(BFS_{t}-BFS_{0}\right)\;\cdot \;C_{s}\;[\mu \varepsilon ]$$

where

○ *S (z)* is the strain at the z coordinate, commonly expressed in microstrain ($\mu \varepsilon =\varepsilon \cdot 10^{-6}$);
○ *BFS_0_* is the Brillouin frequency shift profile acquired at time 0;
○ *BFS_t_* is the Brillouin frequency shift profile acquired at time t;
○ *C_s_* is the transduction coefficient of the optical fiber, equal to 20,000 με/GHz;
○ *με* (microstrain) is the measurement unit of the strain.

The installed optical fiber sensor (Figure 6b) from the inside out is composed of i) a glass fiber with a total diameter of 125 µm, ii) a primary polyamide coating which brings the diameter to 250 µm and iii) a polyvinyl chloride (PVC) coating which brings the final diameter to 900 µm. The percentage of the rock mass strain transferred to the optical fiber sensor varies depending on the glue used, the protective coating and, finally, the bonded length. Parametric studies have shown that the higher the bonded length and the stiffer the coating and the adhesive, the more strain is transferred to the sensor [59,60]. In this work, the effects of such materials on the strain transfer from outside the cable to the sensing-fiber were not computed since the calibration of the strain coefficient (C_ɛ_ = 0.05 MHz/με) performed in laboratory was used. However, several actions were executed in order to minimize the effects of the materials on the strain transfer: i) use of standard widely tested polymers, ii) coatings kept as thin as possible, iii) application of a glue characterized by a suitable value of the elastic modulus, and iv) implementation of a strong bonding between rock mass and sensors. To perform the temperature discrimination, two independent cables were installed parallel to each other. The cable for the strain measurement (500 linear meters long) was reinforced with strands of 316 stainless steel, with a diameter of 0.5 mm, and glued to the structure. The temperature compensation cable (additional 500 linear meters of length) consists of a tube containing the fiber and a gel to improve the heat exchange. This cable is arranged in parallel to the one for the strain measurement and provides only the temperature profile (the fiber inside this second cable is unconstrained).

**Figure 7:**
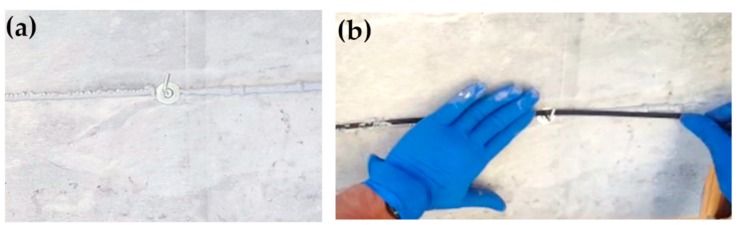
(**a**) Adhesive resin layer applied on the buttress along the path designed for optical fibers. (**b**) Positioning of the sensor cable on the glue layer.

**Figure 8:**
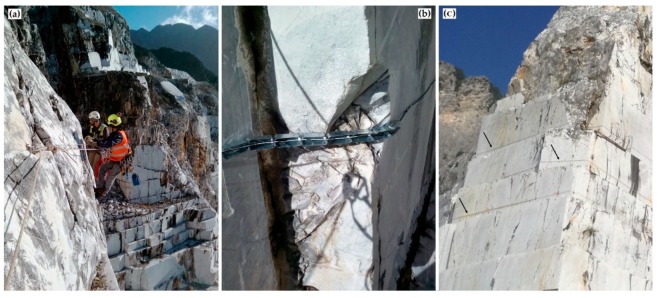
(**a**) Installation of the optical fiber on the marble buttress carried out by specialized climbing workers. (**b**) Detail of a special optical fiber setting, reinforced by metal elements, in an area characterized by artificial rock cut and fracturing. (**c**) The black arrows indicate the sensors (light grey lines) glued to the buttress.

The position of the optical fiber was designed by trying to intercept as many rock mass discontinuities as possible and following the location of topographic measurement prisms and geotechnical sensors on the quarry walls. Two different phases, pre-installation of the witness wire and installation of the effective sensor cable, were carried out by specialized climbing workers (Figure 7 and Figure 8). In particular, the pre-installation phase took place through the installation of mechanical hooks and the engagement of the witness wire. Afterwards, the witness thread was removed, and a layer of “Sikaflex®-11 FC+”, adhesive resin (Figure 7a), based on polyurethane and characterized by a Secant Modulus of Elasticity of ~0.60 N/mm^2^ (after 28 days) (+23 °C) (ISO 8339), was applied through a special compressed air gun. Polyurethane adhesives are “structural adhesives” which can be used to join very different types of materials together with a long-lasting and strong bond. In particular, “Sikaflex® - 11 FC+” is an adhesive commonly and widely used in several works on optical fibers [61,62]. The sensor cable was placed on the adhesive and secured to metal plugs (Figure 7b). The cable was then covered with glue to improve adherence to the surface and to protect DOFS from both meteoric events and ultraviolet rays (Figure 8c). Since DOFS installation cannot have right angles or smaller, some different setting was necessary in zones characterized by artificial rock cuts and fracturing. Figure 8b, as an example, shows a typical setting adopted in these situations.

## 3. Results

### 3.1. Deliverables from UAV Photogrammetry 

**Figure 9:**
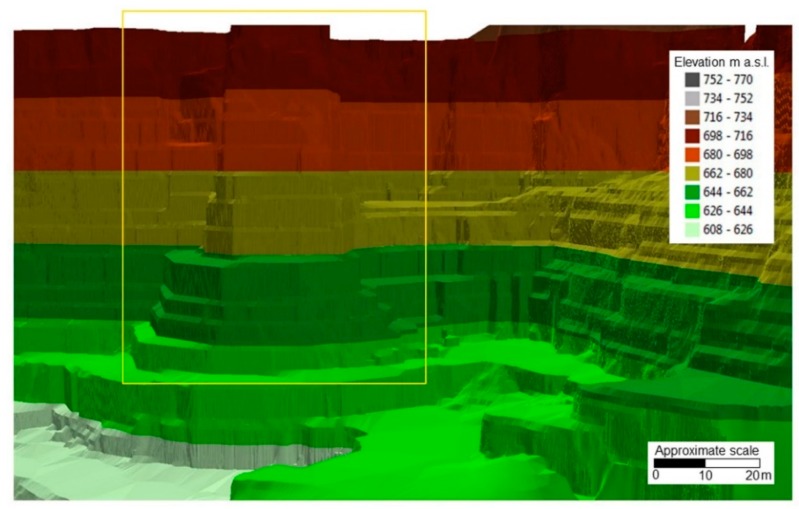
Three-dimensional representation of the elevation through a TIN (Triangulated Irregular Network) model. The yellow rectangle highlights the marble buttress area.

**Figure 10:**
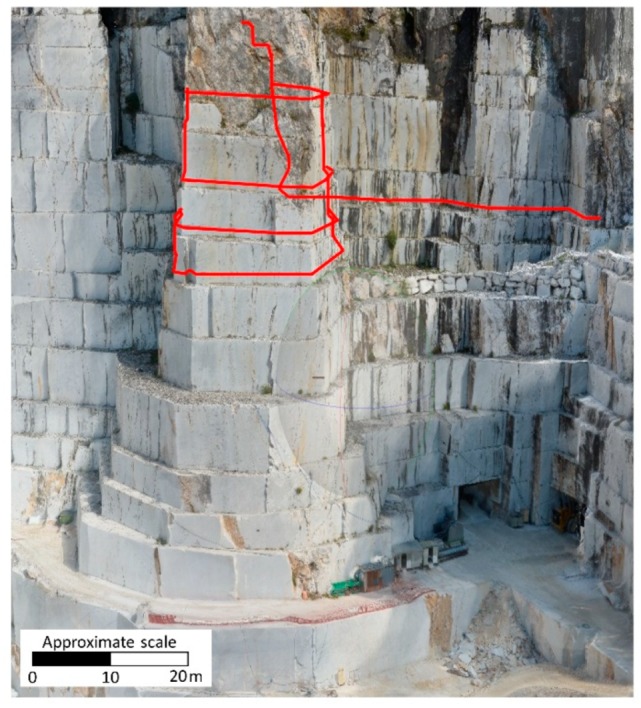
Textured 3D polygonal mesh of the marble buttress with the DOFS spatial distribution highlighted in red.

The photogrammetric processing led to the creation of a 3D dense point cloud, a DEM of the investigated area (GSD of 5.72 cm/pix), shown in Figure 9, and a textured 3D polygonal mesh type model (Figure 10). The polyline representing the spatial distribution of the optical fiber cable was discretized and analyzed in a GIS (Geographic Information System) environment through ESRI^TM^ ArcGIS Pro software. This operation was useful as a preparatory step for the analysis of displacements and strain. In fact, the vectorization and georeferencing of DOFS line allow to i) detect the exact location of deformation data and, ii) make it comparable to contemporaneous information coming from the topographic and geotechnical monitoring systems.

### 3.2. Data from RTS

The measurements recorded by the RTS are represented hereunder through graphs of time versus the slope distance (defined as the distance in a straight line in the space between two points). 

In particular, the RTS displacement values are averaged on a mobile average line over a period of 24 h and filtered by choosing the same hour (at 00:00 h).

**Figure 11:**
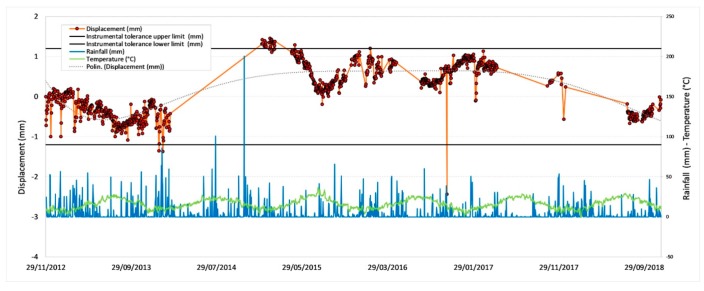
Displacement recorded by the RTS at the measurement point G02 compared to daily average rainfall and temperature. The black lines indicate the instrumental tolerance threshold values.

**Figure 12:**
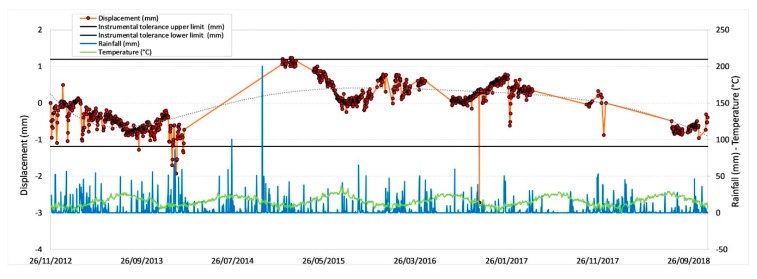
Displacement recorded by the RTS at the measurement point G15.

**Figure 13:**
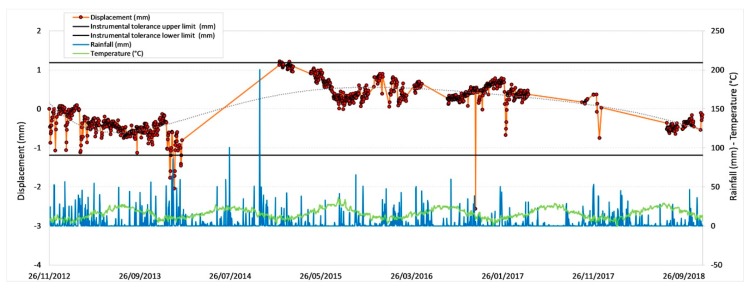
Displacement recorded by the RTS at the measurement point G17.

**Figure 14:**
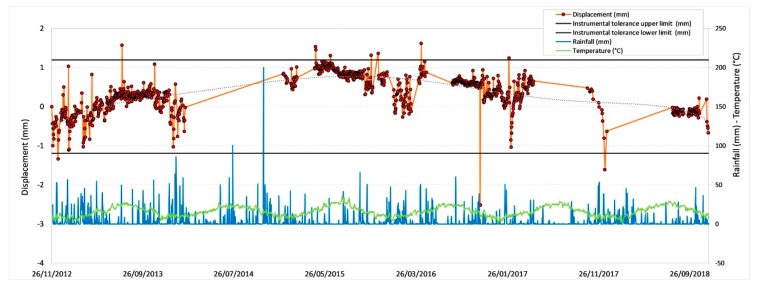
Displacement recorded by the RTS at the measurement point P07.

**Figure 15:**
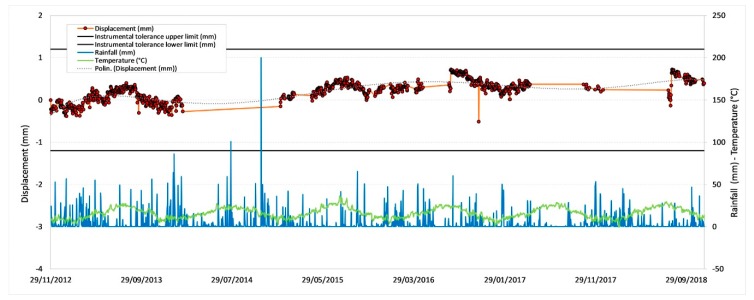
Displacement recorded by the RTS at the reference point R01.

**Figure 16:**
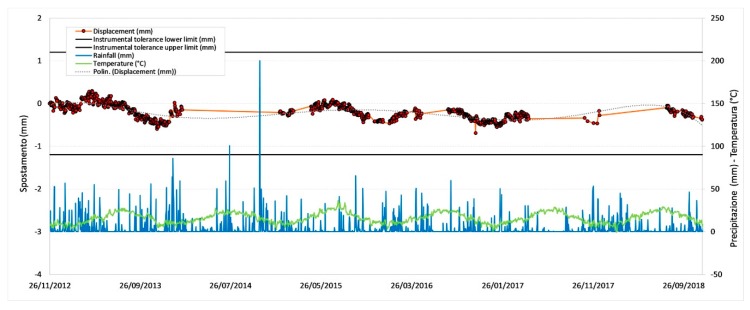
Displacement recorded by the RTS at the reference point R02.

Figure 11, Figure 12, Figure 13 and Figure 14 illustrate the results obtained from some measurement points located near the optical fiber installation. Figure 15 and Figure 16 show the displacement time series of two reference prisms. The time span analyzed was from 2012 to 2018. 

The displacement recorded in every prism was correlated to the daily rainfall and the average daily temperature. The graphs also show the RTS instrumental tolerance threshold values as calculated for the individual prisms taking into consideration the instrumental angular accuracy and their distance from the RTS; these threshold values vary from ± 0.55 mm to ± 1.62 mm [15].

The meteorological data, useful to determine the environmental and climate conditions in the various measurement days, and from these to make the necessary deductions on observed displacements, were provided by the “Carrara Fossola” meteorological station [63].

As can be seen from the graphs in Figure 11, Figure 12, Figure 13, Figure 14, Figure 15 and Figure 16, the RTS suffered periodic malfunctions and phases of inactivity during the whole 2012-2018 timespan. Moreover, this paper aimed at analyzing results that overlap with the available DOFS data. The availability of contemporaneous RTS and optical fiber measurements is limited to the summer and autumn seasons of 2018 and, more specifically to the time interval between 30/07/2018 and 28/11/2018 (about 4 months), with two interruption periods from 06/09/2018 to 22/09/2018 and from 30/10/2018 to 22/11/2018. The interruption periods were related to technical issues.

The analysis of the previous graphs (Figure 11, Figure 12, Figure 13, Figure 14, Figure 15 and Figure 16) suggests a substantial difference in the displacement recorded by the RTS between reference (Figure 15 and Figure 16 ) and measurement points (Figure 11, Figure 12, Figure 13 and Figure 14) linked to the absence, in the first case, of anomalous values. The peaks of measurement points, which bring the associate prism closer to the observation point (i.e., the position of the RTS), occur in conjunction with thunderstorms. All the measurement points, installed in different positions with each other, show this very similar trend, suggesting an almost unanimous behavior of the entire marble structure [15]. Even if the anomalous values exceed the instrumental thresholds, these are values of slight entity (<3 mm) that do not represent a particularly critical situation, as demonstrated by the results from other sensors (as discussed later) and reality, since not rockfall occurred in the whole monitored time span. 

Moreover, there is a generalized sinusoidal trend that can be related to the thermal response of marble slopes to the temperature variation. In fact, on an annual scale, it is possible to see that the displacement values are characterized by a decrease, at the beginning of the warm season, and by an increase with the arrival of the cold season. This behavior of the marble, known as thermoclasty, is most likely related to the anisotropy of calcite mineral, which tends to expand and contract in directions constrained by crystallographic axes [15,64,65,66]. The phenomenon is probably encouraged by different elements: i) the water infiltration within the rock discontinuities during the rain events; this can increase the inner pressure with a consequent growth in volume and expansion of the joints, thus creating a general stress that has repercussions on the whole buttress; ii) the site morphology and iii) the stress reduction due to both mining and natural causes (emersion condition linked to the geology of the area).

### 3.3. Data from the Geotechnical Monitoring System

**Figure 17:**
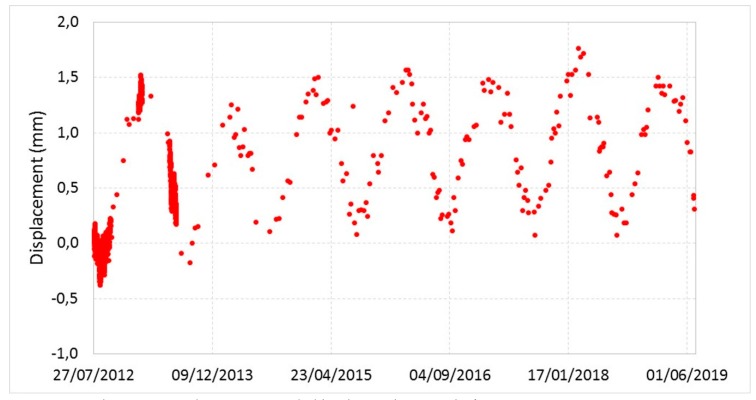
Displacement recorded by the crackmeter FS4 from 2012 to 2019.

**Figure 18:**
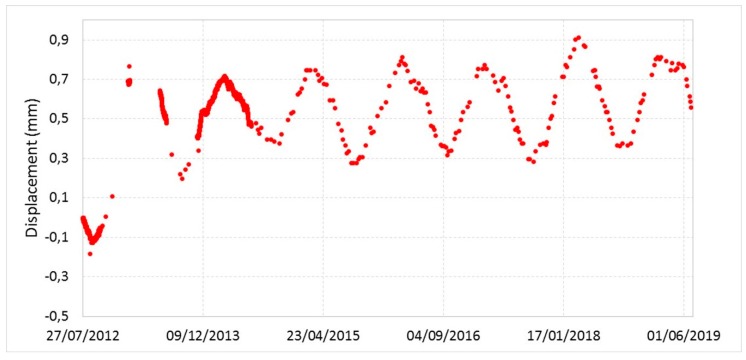
Displacement recorded by the extensometer ES3B1, 6 m long sensor, from 2012 to 2019.

The measurements recorded by the geotechnical sensors in the 2012-2019 time period (some examples are given in Figure 17 and Figure 18 for crackmeter FS4 and extensometer ES3B1, respectively) confirm the general stability of the monitored site. The graphs show sinusoidal symmetrical trends that are yearly replicated with a good approximation. In particular, the values of the observed quantities reach the local minimums during the summer season and the local maximums in winter. This behavior may be attributed to the summer thermal expansion which tends to close fractures. On the contrary, in winter, joint aperture increases for the absence of rock thermal expansion and the presence of water and ice within fractures. Nothing particularly different from previous years was recorded during 2018. The deformation quantities confirm the absence of significant movements of the rock mass [43,44].

### 3.4. Data from DOFS

**Figure 19:**
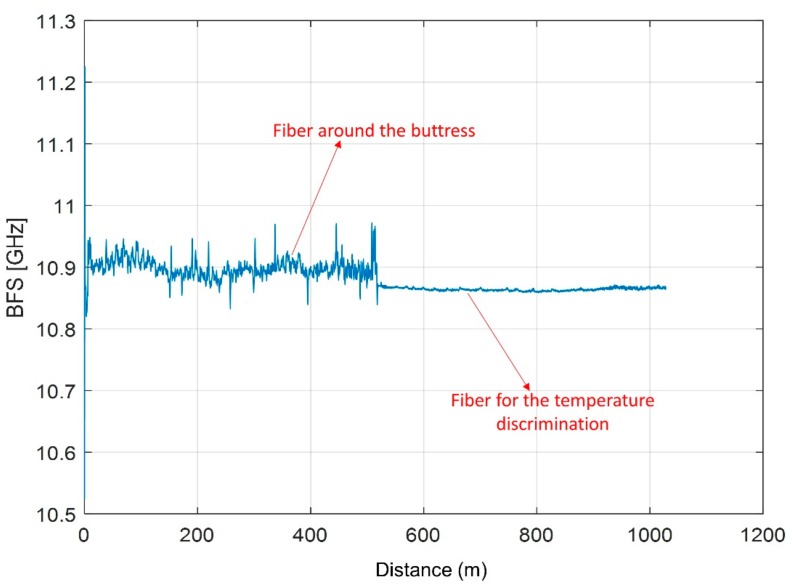
BFS profile acquired on 4 January 2018 (first measurement).

In the Lorano marble quarry, BOTDA type measurements were performed at a spatial resolution of 20 cm. The first acquisition was executed on 4 January 2018 (Figure 19). 

The first 500 m of the profile (Figure 19) correspond to the fiber sensor fixed around the buttress, and present the typical oscillations deriving from the deformations associated with the gluing. The profile continues for another 500 m, maintaining, in this second part, a much more stable trend which is typical of the fibers used for the temperature discrimination [67].

**Figure 20:**
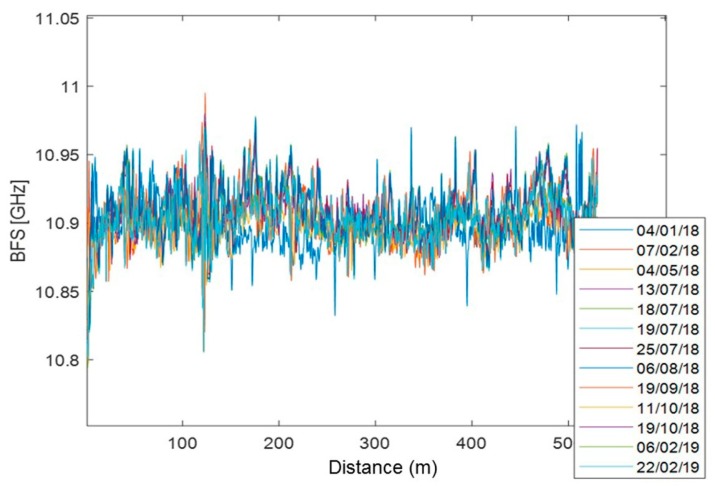
BFS profiles acquired from January 2018 to February 2019.

From January 2018 to February 2019, there are no significant changes in the BFS values that remain within ±0.1 GHz (Figure 20). The only evident deviation concerns the first acquisition (carried out on 4 January 2018) which, particularly in the points around 120 m and 260 m distant from the origin, shows slightly lower BFS values than the other acquisition dates. Since, in these points of the buttress, the other measurement sensors (RTS and geotechnical) were consistent with each other, it was assumed that this variation is due to the adjustment of the deformation state of the fiber following its recent installation. Therefore, for the calculation of the strain, it was decided to exclude the measurement of 4 January 2018, thus assuming as the first value that of 7 February 2018, after a certain adhesive resin hardening. 

**Figure 21:**
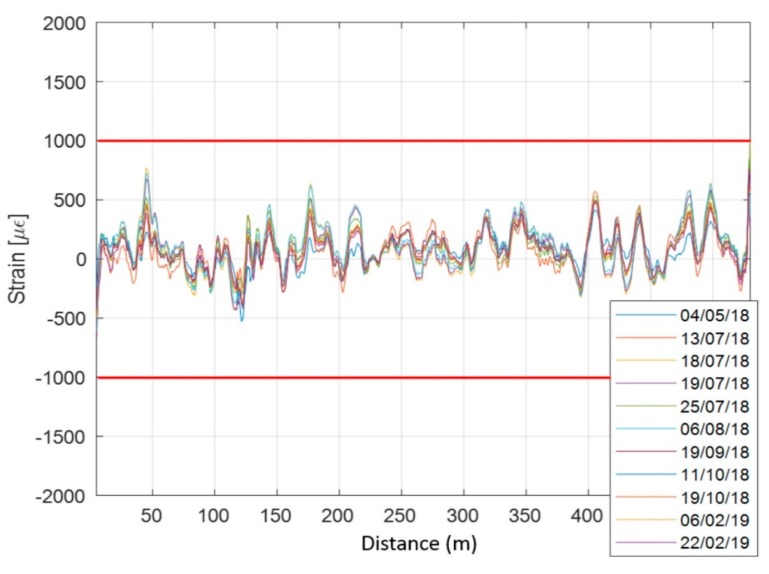
DOFS strain profiles along the marble buttress from January 2018 to February 2019 and alarm thresholds (red lines).

Strain was calculated by subtracting, for each measurement position (every 20 cm), the value of BFS measured on 7 February 2018 to that measured later and multiplying the variation of BFS thus obtained by the transduction coefficient equal to 20,000 με/GHz [51]. The obtained strain data was finally filtered using a moving average filter over 51 points (Figure 21), equivalent to a spatial resolution of 10 m.

As can be seen from Figure 21, the most significant deformations appear during the summer months with a reversible behavior. The strain values are relatively low if compared to the standard deviation of each profile (approximately 200 με). As suggested by the literature [68], it was decided to set the alarm threshold as a multiple of the standard deviation of the strain values. The obtained value of standard deviation suggests for the authors an alarm threshold for deformations five times greater (i.e., equal to 1000 με). At the Rocks Mechanics Laboratory of CGT, compressive strength tests were performed on marble samples from the site test of this work. The results of these analyses show an Elasticity Modulus equal to 60 GPa and a Compressive Strength ($\sigma_{u}$) value equal to 87 MPa. Similar values were confirmed by the literature data [69]. Considering the measured value of the Elasticity Modulus, the stress value corresponding to the strain of 1000 με is given by

(4) $$\sigma_{\mathrm{calc}}=\mathrm{strain}\;\left(\mu \varepsilon \right)\;\cdot 10^{-6}\cdot E\;≅59\;\mathrm{MPa}$$

Since $\sigma_{\mathrm{calc}}<{\;\sigma }_{u}\;$, the value of 1000 με was considered a suitable and precautionary alarm threshold value for the strain values in the Lorano marble quarry. Figure 21 shows how this threshold value was not reached in any position throughout the period of the DOFS monitoring survey.

**Figure 22:**
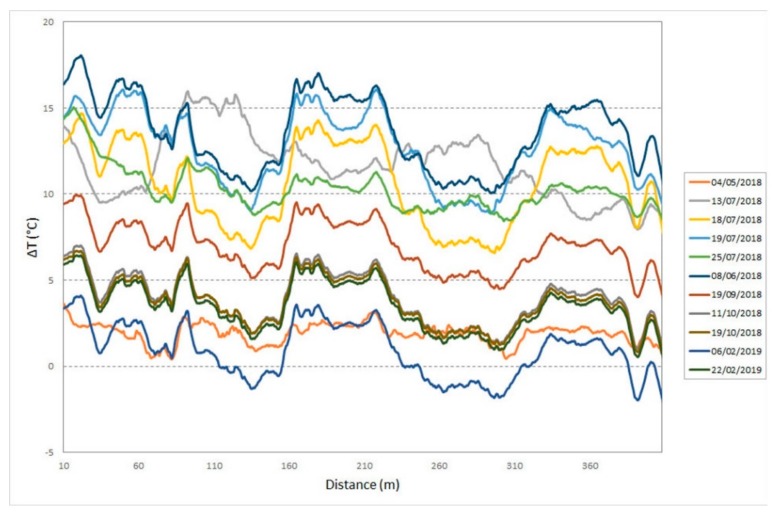
DOFS temperature variation profiles along the marble buttress from January 2018 to February 2019.

Profiles of temperature variation (Figure 22) were obtained starting from the BFS data and using 7 February 2018 as the reference measure. As in the case of strain, the temperature data were filtered using a moving average filter over 51 points (still achieving a spatial resolution of 10 m). The graph shows the natural difference between the temperature measurements taken in autumn and winter compared to those taken in spring and summer. The temperature data detected by optical fiber, in fact, vary between −10 °C and + 20 °C, as result of the natural seasonal variations in the case of an open pit.

**Figure 23:**
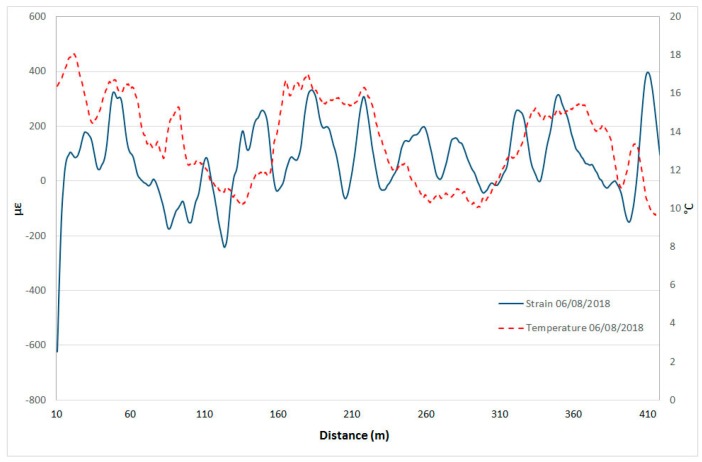
Example of comparison of strain and temperature profiles in summer.

**Figure 24:**
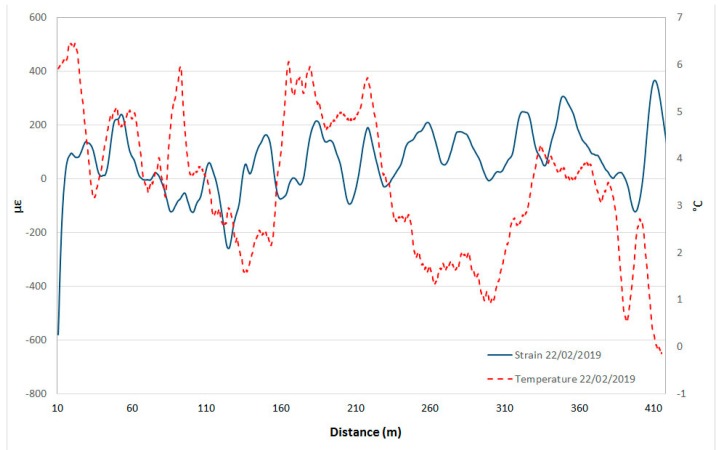
Example of comparison of strain and temperature profiles in winter.

Figure 23 and Figure 24 show two examples of the comparison between strain and temperature at two individual dates, one for the summer (Figure 23) and the other for the winter season (Figure 24). The graphs demonstrate how higher temperatures correspond to greater strain. It is important to underline that these strain values are always lower than the alarm threshold values of Figure 21.

## 4. Discussion

During the phase of DOFS data acquisition, some critical issues emerged due to both the complex morphology of the study area and the fragility of the instrumental components, both in terms of fiber cable and control unit. The intersection between fiber optics and rock mass asperities, in fact, led to several phenomena of failure of the sensor cable. However, these breaks were promptly repaired using a more robust protective sheath coupled with aluminum channels and steel cables (Figure 8b). Moreover, a damage to the OSD-1 system backup batteries and the surrounding components caused the interruption of data recording for over 3 months (i.e., from March to June 2018) waiting for the replacement parts. 

Nevertheless, the data acquisition time span lasts in total for about one year, giving the possibility to analyze the obtained results, compare them with data from different monitoring techniques and highlight strengths and weaknesses of DOFS in such operative conditions. 

Before giving some comments on the obtained results, some considerations about the possible errors due to the uncertainty of the strain transfer from the rock to the strain-sensing fiber must be made. Using the optical fiber as a sensor, it must be considered that the strain of the optical fiber is not that of the monitored structure due to both the glue and the coating. Moreover, because of geological stresses and gravity, the detection of the strain is a function of the fiber orientation with respect to the quarry walls; consequently, the deformation measured by the sensor is a percentage of that of the structure. Experimental studies [59,60] showed that the higher the adhesion length, the greater the strain transfer rate; the higher the shear modulus of the coating layer and the adhesive, the greater the strain transfer rate. In this work, it was attempted to minimize the effects of materials by choosing suitable standard polymers as polyurethane (adhesive layer), polyamide and PVC (coatings) and realizing suitable adhesion lengths. Specific analyses on the effects of the materials were not done since the strain coefficient as calculated in laboratory was used. Basing on this assumption, the uncertainty on the strain measurement is that of the OSD-1 measurement system (i.e., about 20 µε). If, along the entire installed cable, the calibrated strain coefficient is constant (and therefore the strain transfer is also constant), there are no other errors to consider. Obviously, if the installation is faulty, there may be variations from point to point, but it is impossible to take them into account.

**Figure 25:**
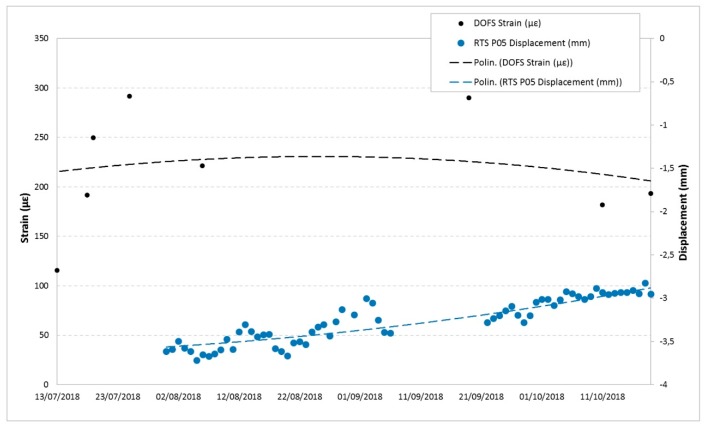
Comparison between the P05 RTS measuring point displacement (data interpreted according to a second order polynomial) and DOFS strain values in the same area.

**Figure 26:**
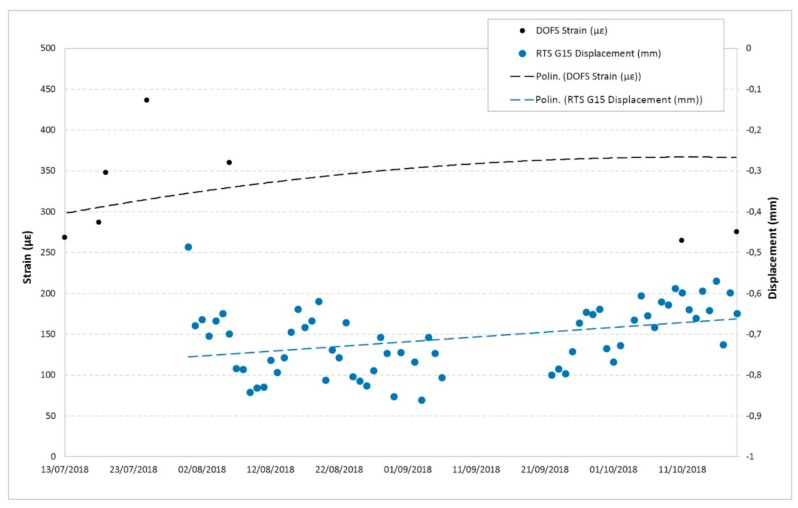
Comparison between the G15 RTS measuring point displacements (data interpreted according to a second order polynomial) and DOFS strain values in the same area.

**Figure 27:**
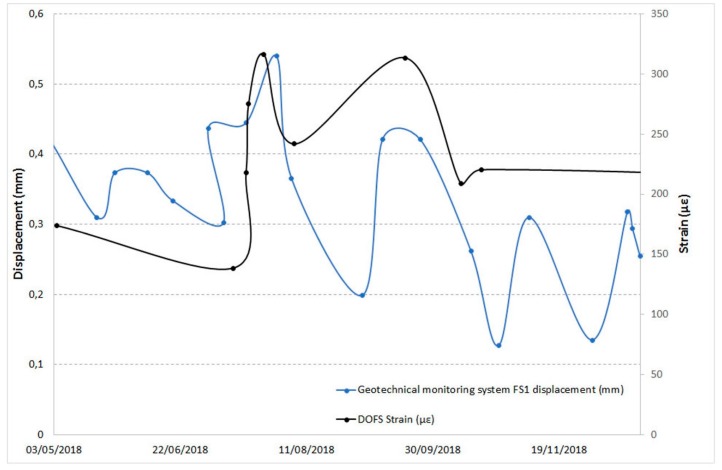
Comparison between the FS1 crackmeter displacements and DOFS strain values in the same area.

**Figure 28:**
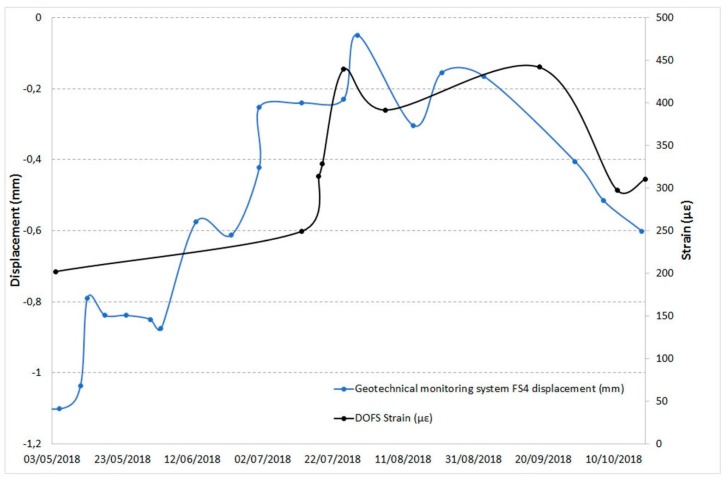
Comparison between the FS4 crackmeter displacements and DOFS strain values in the same area.

**Figure 29:**
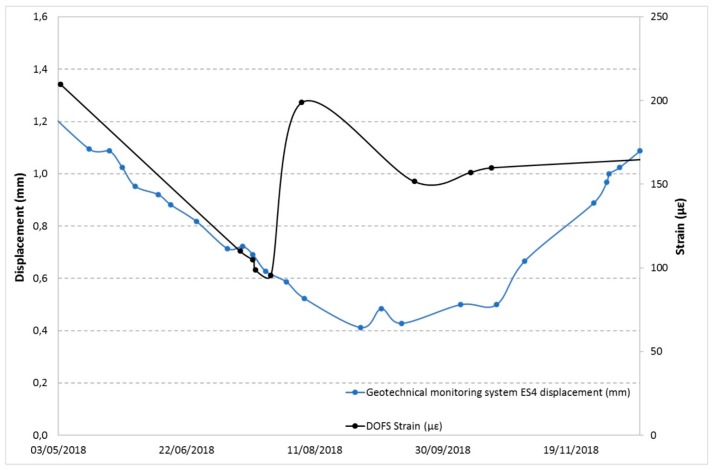
Comparison between the ES4 extensometer displacements and DOFS strain values in the same area.

**Figure 30:**
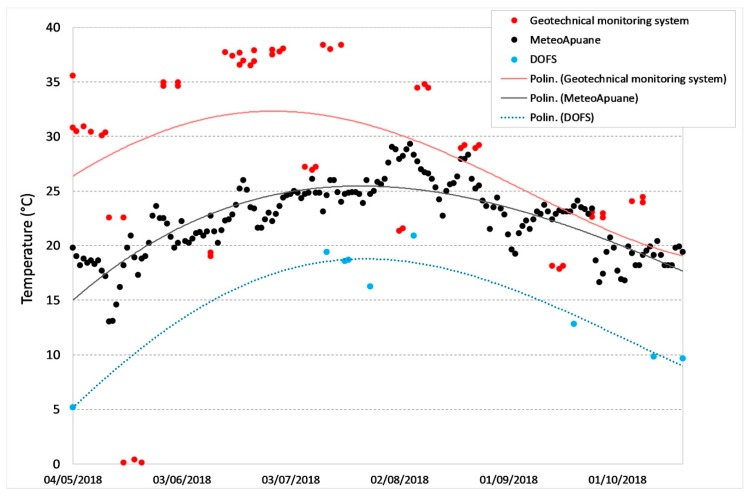
Comparison between the temperature values measured by DOFS, FS1 geotechnical sensor and “Carrara Fossola” weather station. The data were interpreted according to a fifth order polynomial.

Below are some examples of comparison with displacements recorded by the other monitoring techniques in the overlapping time periods. Figure 25 and Figure 26 show the DOFS strain values versus the RTS displacements, Figure 27, Figure 28 and Figure 29 illustrate the DOFS strain values versus the geotechnical monitoring system displacements, and Figure 30 juxtaposes the temperature values measured by DOFS, the geotechnical monitoring system and the “Carrara Fossola” weather station.

At the observed scale, the trends of strain values from DOFS and that from displacements recorded by RTS and geotechnical sensors are very confusing even if quite similar if compared to each other. There is a close correspondence between the trend of the displacement values recorded by the crackmeters and the strain profiles (examples are reported in Figure 27 and Figure 28), characterized by a certain cyclicity and higher values in the warmer months. Figure 29, instead, shows an example of a comparison between the extensometer displacements and the DOFS strain values. Both graphs show a decreasing trend, a local maximum reached in the hottest period (first recorded by the optical fiber) and a rising trend in the last part of the year.

The trends of the average daily temperature values measured by DOFS (average of all values recorded along the DOFS sensor cable), geotechnical monitoring system and weather station (Figure 30) are quite comparable, even if the DOFS values are lower than the others. This may be due to several reasons: first, to the different altitude between the station (55 m a.s.l.) and the marble buttress (about 600-700 m a.s.l.); secondly, to the coating and to the glue necessary to protect fiber optic from both meteoric events and ultraviolet rays. Moreover, the temperature measured by the fiber, resulting from the external temperature (air) and the temperature of the rock where the fiber is placed, vary according to the time of day, the position and the sunlight exposure: there may be shaded areas and zones exposed to the sun along the fiber path. These conditions have their effect in determining the average temperature values along the entire path of the optical fiber.

Despite the short time of testing and small number of results, it can be affirmed that the proposed system has provided a reliable and accurate monitoring measure for active marble open pit deformation control over wide extension.

Conventional monitoring techniques, such as extensometers, crackmeters, and RTS, are capable of detecting the deformation in single points while DOFS can allow to monitor lines and, with suitable installation, surfaces, allowing to improve the accuracy, the completeness and the safety of the whole system.

Moreover, the high accuracy of the data shows that optical fibers can detect precursory signs of failure well before the collapse, paving the way for the development of more effective early warning systems. In this sense, the work presented here had the goal of improving the monitoring activity already implemented in the quarry in such a way as to allow planning of the mining activity development in the short and medium term in compliance with safety standards and in the interest of production. 

The DOFS test for monitoring the Apuan Alps marble quarries is a new experiment. Moreover, due to their distinctive features, such as sub-vertical slopes and walls often jutting out, and tunnels, these extraction sites represent an ideal site for the experimental development of such a monitoring system.

These studies, together with additional data on risk assessment already available for the Lorano marble quarry, represent a great help for the excavation, pre-excavation and post-excavation periods, as well as for other displacement-dependent engineering-geological projects. For example, the DOFS monitoring system can be joined to distinct element numerical modelling to simulate the deformation features of an extraction front and to provide the scientific basis for detecting the early warning signs of a rockfall.

## 5. Conclusions

An innovative monitoring system was implemented by means of DOFS based on BFS on the walls of a marble buttress located in the Lorano quarry, in Italy. The system has been operative for about one year and the obtained results were compared with an integrated topographic-geotechnical monitoring system operative at the extraction site since 2012.

The evaluation of the effectiveness of the DOFS, with reference to their capability of data acquisition, is undoubtedly positive: the system, in fact, despite the encountered difficulties related to both the complex morphology of the area and the fragility of the components, proved to be able to carry out the detection of reliable and very precise BFS, strain and temperature data.

At the test site, BOTDA measurements were performed. The most significant deformations appeared during the summer months with a reversible behavior. The recorded strain values are relatively modest compared to the standard deviation of each profile (approximately equal to 200 µε). This standard deviation value suggests that the choice of an alarm threshold for deformations at least five times greater, that is, equal to 1000 µε, never reached in any position throughout the timespan of the monitoring. This evidence was confirmed by the displacement values found at specific measurement points by the RTS. Since these displacement values fall within or very near to the range defined by the instrumental tolerance threshold, no critical situations were detected, in accordance with results obtained with the optical fiber survey. Moreover, the comparative analysis of all the data acquired by the geotechnical monitoring system confirms the absence of significant displacements of the buttress in the studied time period.

The temperature data detected by optical fiber, variable in the range between −10 °C and + 20 °C, appear to be affected by natural seasonal variations, as it is natural to expect in the case of an open pit.

The obtained results confirm the potential of the DOFS monitoring system to detect and locate any deformation phenomenon that may occur along the fiber path, even in hostile and less hospitable environments like the marble extraction sites. However, some difficulties mainly related to the authors’ inexperience were faced: considering the nature of a monitoring system (need for long-term analysis) and the encountered technical problems, it would be appropriate to continue the experiments in the future in order to increase the amount of the available data. In fact, a more substantial dataset could allow more in-depth assessments to be made regarding the effectiveness of the DOFS monitoring system and its durability.

The installed systems will be used continuing the measures to evaluate the progression of the deformation over time in order to provide useful indications for the realization of a pre-alarm system.

This latter can be a valid tool for guaranteeing safety conditions for quarry workers and planning the actions to be undertaken for the continuation of excavation work in adjacent areas.
